# Evaluation of the Success Rate of Nonsurgical Single Visit Retreatment

**Published:** 2007-07-05

**Authors:** Hengameh Ashraf, Amin Salem Milani, Shahla Shakeri Asadi

**Affiliations:** 1 *Department of Endodontics, Iranian Center for Endodontic Research, Dental School, Shahid Beheshti University of Medical sciences, Tehran, Iran*; 2 *Department of Endodontics, Dental School, Shahid Beheshti University of Medical sciences, Tehran, Iran*; 3 *General Practitioner, Tehran, Iran*

**Keywords:** Retreatment, Single Appointment, Success Rate

## Abstract

**INTRODUCTION:** Advantages of endodontic treatments over tooth extraction have increased the demands for these treatments. Success rate of these treatments is generally lower than the primary root canal therapies. Recently, single visit treatments have gained more popularity. But, the influence of completing retreatment in a single appointment on success of the treatment is still a controversy. The aim of this study was evaluating the most common causes of failures and determining the success rate of completing retreatments in single appointment.

**MATERIALS AND METHODS:** Hundred and twenty four patients who had single appointment retreatment within past 4 years in postgraduate ward of Shahid Beheshti Dental School were selected and clinical and radiographic examinations were carried out. The data were analyzed by SPSS using Chi-square and Exact Fisher tests.

**RESULTS:** Success rate and uncertain cases were shown to be 50.7% and 34.2%, respectively and failure rate was 15.1%. Presence of periradicular lesion or history of swelling prior to retreatment had significant effect on the success of single visit retreatments (*p*<0.001). Another important finding of the study was that the success rate of retreatments was significantly higher in cases referred for restorative purposes compared with retreatments for nonrestorative purposes (*p*<0.001).

**CONCLUSION:** The overall success rate of single appointment retreatments is up to 84.9% which is considerably higher in cases referred for restorative purposes. So based on the results of this study single appointment retreatment of symptom less teeth is recommended.

## INTRODUCTION

Advantages of endodontic treatments over tooth extraction have increased the demands for these treatments. Success rate of these treatments has been reported between 86% and 95% (1). So even in best possible conditions there is about 5 % failure rate.

There are many reasons for failures of endodontic treatments including poor canal preparation and obturation, iatrogenic procedural errors such as perforations, overfilled or missed canals (2-3). An examination of failed cases from the Washington study (3) showed that over two third of these failures were related to incomplete cleaning and obturation of root canals. Harty *et al.* have also reported that the majority of nonsurgical endodontic procedures that fail do so because of inadequate apical seal (4). The single factor most of these failures have in common is the presence of microorganisms. So, locating the source of infection is the key factor in management of failed root canal therapies (5).

The reported success rate of retreatments of failures is between 40 to 100%, generally lower than the primary root canal treatments (6-10) which may be attributed to poor access to original root canal system due to modified canal anatomy and difficulties in eradication of resistant bacterial species in root canals. Even so, novel techniques and equipments together with high quality education systems have somehow increased the success rate of these treatments (11).

**Table 1:** Success, Uncertain and failure rate relative to some procedural errors

	**S**	**U**	**F**	**Sig.**
Overfilled	2	6	0	-
Underfilled	61	30	17	
Proper length	11	14	5	
Separated instrument	4	3	0	-
Without separated instrument	70	47	22	
Poor cleaning & shaping	64	46	15	**a**
Adequate cleaning & shaping	10	4	7	
Missed canal	6	4	4	-
Without missing	68	46	18	
Perforation	0	4	4	**a**
Without perforation	74	46	18	
Ledges	10	12	12	**a**
Without ledges	64	38	10	
Non Gutta-percha filling	10	10	0	**a**
Only Gutta-percha filling	64	40	22	

**S:** Success, **U:** Uncertain, **F:** Failure, **a*:*** Significance (*P*<0.05)

Eradication of infection is the primary goal of these treatments which is achieved by chemical disinfection and mechanical debridement of root canal system followed by intracanal medication (12-15).

Recently, single visit treatments have gained more popularity. Completing the treatment in a single appointment has many advantages including reduction in treatment time and cost, lower risk of microleakage and recontamination of root canals between appointments which is more common in multiple visit treatments (16-22).

The aim of this study was evaluation of the most common causes of failures and success rate of completing retreatments in single appointment.

## MATERIALS AND METHODS

This analytic-descriptive study was carried out using patients' documentary, clinical and radiographic examination on 124 patients referred to Shahid Beheshti Dental School in 2002 until 2006 for retreatment.

Hundred and twenty four patients who received single appointment retreatment within past 4 years in postgraduate ward were selected and called for follow up; clinical and radiographic examinations were carried out. All the examinations were done by a same examiner. The data were collected using patients' documents, clinical and radiographic examinations.

History of periapical lesions, swelling, resorption, procedural errors such as inadequate cleaning and shaping, ledge and transportation, instrument separation, missed canals, poor root filling quality or length control, all were collected from the documents.

Clinical examination didn’t reveal any pathologic condition, sign or symptom of failure such as swelling, sinus tract, sensitivity to percussion or palpation and mobility.

Periapical radiographies provided by paralleling technique were compared with pretreatment radiographies by direct visual inspection (23).

Success and failure were judged according to the following criteria (23-24):

**A)** Success:

- No sign or symptom in clinical examination

- No radiographically detectable periradicular lesion

**B)** Uncertain:

- No sign or symptom in clinical examination

- Slightly reduced size of periradicular radiolucency in radiographic examination

**C)** Failure:

- Presence of pathologic signs or symptoms in clinical examination

- History of endodontic surgery or extraction following treatment

- Development of periapical radiolucency in teeth without any lesion in preoperative radiographies or increasing sized radiolucency in follow up radiographies compared to preoperatives.

The chief complaint of the patients or the main reasons for referral were extrapolated from documentary and categorized as restorative and nonrestorative needs.

Restorative needs included cases requiring new restorations due to failed previous restorative treatment or any other reasons.

Nonrestorative reasons included other indications for retreatments such as pain, swelling, mobility, and any other sign or symptom of failure.

The data were analyzed by SPSS ver.14 using Chi-square and Exact Fisher tests.

## RESULTS

Hundred and twenty four patients (146 teeth) included in the study were 55.4 % (n=69) female and 44.6 % (n=55) male with a mean age of 39.9 years.

**Table 2:** Success, Uncertain and failure rate relative to the presence of periradicular lesion, history of swelling, and main reason of referral

	**S**	**U**	**F**	**Sig.**
History of swelling	8	16	18	**a**
Without history of swelling	66	34	4	
Periapical lesion	45	50	22	**a**
Without periapical lesion	29	0	0	
Restorative	31	26	2	**a**
Non Restorative	14	24	20	

**S:** Success, **U:** Uncertain, **F:** Failure, **a*:*** Significance (*P*<0.05

The success and failure rate related to some common procedural errors has been demonstrated in Table 1.

The influence of the history of swelling and periapical lesion on success or failure has been displayed in Table 2. In this table, the restorative and nonrestorative reasons have been separated and their influence on retreatment success has been demonstrated.

## DISCUSSION

In a thorough search of published literature review on the success of nonsurgical retreatments Paik *et al.* assigned level of evidence to these published data and concluded that only few high level studies have been published in the past 34 years related to the success and failure of endodontic retreatments (10). In our study, Success rate and uncertain cases were shown to be 50.7% and 34.2%, respectively and failure rate was 15.1%. Several studies have reported similar success rate for single and multiple visit treatments (16, 18). Peters and Wesselink reported a similar 81% and 71% success rate of single visit versus two appointment root canal treatments, respectively (19). Jurcak *et al.* reported high success rate of single visit treatments of about 89% on 167 teeth (20). Trope *et al.* found high success rate of single appointment root canal treatment in teeth without apical periodontitis (21). However, in a recent study on 218 patients the incidence of postoperative pain was lower after the two visit RCT compared to single visit treatments (22).

The data displayed in Table 1 show that some factors such as non gutta-percha containing filling materials or poor shaping and filling have influenced the success rate less than some other procedural errors such as perforation or ledges. It can be concluded that the more damage to and alteration of the original anatomy of the canal during primary RCT, the more failure rate of the retreatment will be expected. These results are in harmony with the conclusions of the study carried out recently by Fabio *et al.* in which alteration of the original anatomy of the canal are reported to be a major influencing factor on retreatment success (25).

History of swelling has a significant effect on the success of the single visit retreatments (Table 2) descending the success rate from 63.4-96.2% to 19%.

These apparent differences may be attributed to inability of eradicating resistant microorganisms from infective canals in single appointment without intracanal medications and also inability of complete preobturation drying of canal.

According to the presented data in (Table 2), presence of periradicular lesion is another factor that reduces the success rate from 100% to 38.4%. These results are also in harmony with other studies on this topic which have reported 89-100% success rate for teeth without apical lesions and 40-88% when there are apical lesions (3,7-9).

Another important finding of the study is that success rate of retreatments with periradicular lesions was 52.5-96.6% in cases referred for restorative purposes whilst only 24.1% of the retreatments for nonrestorative needs was successful.

It may be concluded that single visit retreatments were more successful in symptom- less cases requiring endodontic treatment just for restorative purposes or low quality RCTs compared to cases with clinical signs and symptoms such as pain and swelling.

A recent study by Yoldas *et al.* also found more success in pain control by two visit versus single visit retreatments (22). Other researches in this field have also shown 63.8-98% success rate in retreatment of cases referred for restorative needs (8,9,26).

## CONCLUSION

The overall success rate of single appointment retreatments may be up to 84.9% which is considerably higher in cases referred for restorative purposes. So, based on the results of this study single visit retreatment of symptom- less teeth is recommended.
